# Comparing various scoring system for predicting overall survival according to treatment modalities in hepatocellular carcinoma focused on Platelet-albumin-bilirubin (PALBI) and albumin-bilirubin (ALBI) grade: A nationwide cohort study

**DOI:** 10.1371/journal.pone.0216173

**Published:** 2019-05-02

**Authors:** Soon Kyu Lee, Myeong Jun Song, Seok Hwan Kim, Misun Park

**Affiliations:** 1 Division of Hepatology, Department of Internal Medicine, College of Medicine, The Catholic University of Korea, Seoul, Republic of Korea; 2 Korean Liver Cancer Study Group, Seoul, Republic of Korea; 3 Ministry of Health and Welfare, Korea Central Cancer Registry, Goyang-Si, Republic of Korea; 4 Department of Biostatics, Clinical Research Coordinating Center, College of Medicine, The Catholic University of Korea, Seoul, Republic of Korea; Kaohsiung Medical University Chung Ho Memorial Hospital, TAIWAN

## Abstract

**Background:**

We evaluated the ability of various grading scales including platelet-albumin-bilirubin (PALBI) and albumin-bilirubin (ALBI) grades to predict overall survival (OS) according to treatment modality in patients with hepatocellular carcinoma (HCC).

**Methods:**

The cohort of 6,669 patients with HCC was selected randomly from the 2008–2012 national cohort of the Korean Central Cancer Registry. The OS of 6,507 of these patients was evaluated using the Child-Turcotte-Pugh (CTP) class, Model for End-stage Liver Disease (MELD) score, and ALBI and PALBI grades.

**Results:**

The patient’s mean age was 59.7 years. The most patients were hepatitis B virus (63.4%) and CTP class A (71.8%). The median OS durations of PALBI grade1 (38.4%), grade2 (33.2%), and grade3 (28.4%) patients were 81, 30, and 5 months, respectively (*P*<0.001). The PALBI grade had a larger area under the receiver operator characteristic curve (AUC) than did the CTP class, MELD score, and ALBI grade (overall AUC: 0.675 vs. 0.633, 0.645, and 0.642, respectively; P < 0.001). Moreover, the PALBI and ALBI grades enabled sub-classification of CTP A patients (*P* < 0.001). In a multivariate analysis, the PALBI and ALBI grades were significant risk factors for OS (P < 0.05). According to treatment modality, the PALBI grade was predictive of OS in patients receiving transarterial chemoembolization or supportive care. The ALBI grade was predictive of OS in patients undergoing surgical resection or radiofrequency ablation.

**Conclusion:**

The PALBI and ALBI grades are more reliable for accessing liver function and predicting OS in patients with HCC. Moreover, according to treatment modality, appropriate use of the ALBI and PALBI grades will enable accurate determination of the prognosis of patients with HCC.

## Introduction

Hepatocellular carcinoma (HCC) is a common type of cancer and a major cause of death worldwide [1]. Despite the development of new therapies, HCC remains difficult to treat because it typically occurs in advanced liver disease or cirrhosis [2]. Treatment decisions and prognosis prediction for patients with HCC are based on performance status, liver function, and tumor burden [3]. Thus, the evaluation of liver function is important in the management of HCC. The Child-Turcotte-Pugh (CTP) class and the Model for End-stage Liver Disease (MELD) score are used widely to assess liver function [4].

The CTP class was developed to predict mortality in patients undergoing surgery for portal hypertension especially variceal bleeding [5, 6], and is currently used to estimate the liver functional reserve and to predict overall survival (OS) in patients with HCC. The CTP class has several limitations: (1) it includes subjective factors, such as ascites and hepatic encephalopathy; (2) each variable is assigned the same weight; (3) some of the variables included, such as ascites and the albumin level, may be related; and (4) arbitrary cut-off levels result in the assignment of the same score to patients with different bilirubin levels [4, 7]. In addition, the inability to discriminate liver function and clinical outcomes among patients with HCC and well-preserved liver function is a major drawback of the CTP class system, as the majority of patients with HCC are of CTP class A [8, 9]. The MELD score is reliable for stratification of the risk of dropout in patients with HCC [10]. However, this score has limitations when applied to patients with less-severe HCC, and has been evaluated only with those awaiting liver transplantation (LT) with “exception” points [4, 9, 11]. Therefore, a new index of the liver functional reserve is needed.

The albumin-bilirubin (ALBI) grade and platelet-albumin-bilirubin (PALBI) grade were introduced to assess liver function in patients with HCC [9]. The ALBI grade is based on laboratory findings, together with the albumin and bilirubin levels, and may be reliable for the assessment of liver function in patients with HCC [12–14]. The PALBI grade, based on the ALBI grade, was developed to reflect the effect of portal hypertension. As a surrogate for portal hypertension, it includes consideration of the platelet count [15]. However, there was no study for evaluating the highest performance scoring system including PALBI and ALBI grades in each treatment modalities. Therefore, we investigated the prognostic performance of the ALBI grade, PALBI grade, CTP class, and MELD score in Korean patients with HCC according to treatment modality.

## Materials and methods

The Institutional Review Board of the Catholic University of Korea approved this study (DC17RESI097) and waived the requirement for informed consent. This study was conducted in accordance with the Declaration of Helsinki.

### Patients

**Fig 1:**
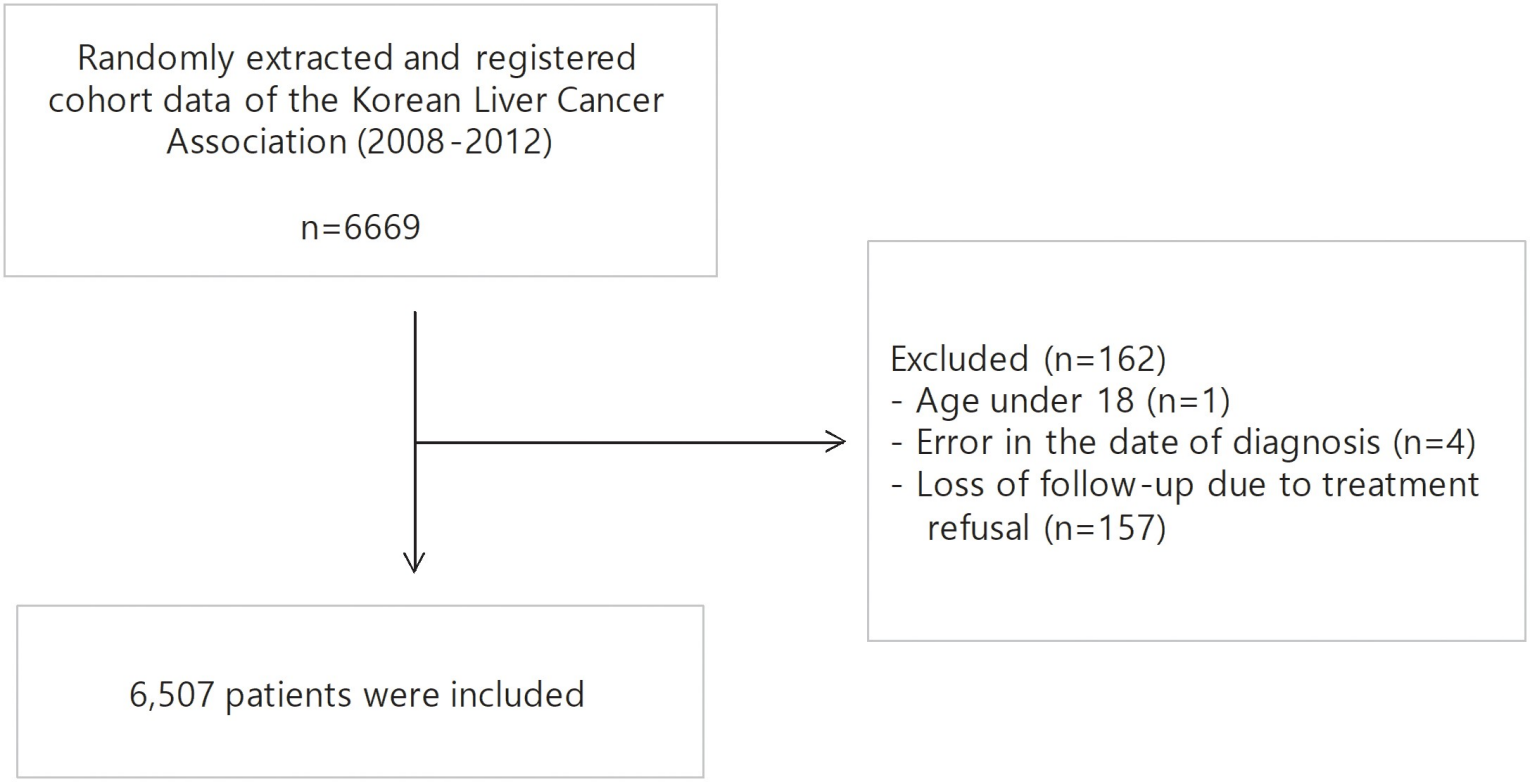
Flow chart of this study.

The South Korean Ministry of Health and Welfare has maintained the Korean Central Cancer Registry (KCCR) since 1980. All new diagnosed cancer cases are registered in the database of KCCR. The Korean Liver Cancer Association (KLCA) has been randomly extracted and registered HCC cohort data from the database of KCCR. In this study, 6,669 patients who are registered in KLCA cohort with newly diagnosed HCC between 2008 and 2012 are enrolled. Of them, 162 patients were excluded for the following reasons: (1) age < 18 years (*n* = 1), (2) erroneous date of HCC diagnosis (*n* = 4), and (3) loss to follow-up due to treatment refusal (*n* = 157). Finally, 6,507 patients with HCC were included in this study (Fig 1). Survival data were obtained from the records of hospitals and/or the National Health Insurance Service of Korea through December 2016.

### Clinical and laboratory data

HCC was diagnosed according to the Korean Liver Cancer Study Group (KLCSG) and National Cancer Center (NCC) of Korea guidelines as follows: (1) pathological diagnosis; (2) diagnosis by one or two imaging modalities with ≥1 cm liver nodules in high-risk patients, such as those with hepatitis B virus (HBV) or hepatitis C virus (HCV) infection or liver cirrhosis; or (3) diagnosis by two or more imaging modalities with <1 cm liver nodules and a steadily increasing serum alpha-fetoprotein (AFP) level in high-risk patients. Diagnosis by imaging modalities was based on the following hallmarks of HCC: hypervascularity in the arterial phase and washout in the portal or delayed phase of dynamic computed tomography, dynamic magnetic resonance imaging (MRI), or gadolinium-ethoxybenzyl-diethylenetriamine pentaacetic acid–enhanced MRI [16].

The CTP class and MELD score were calculated at the time of diagnosis (Table 1) [17]. The MELD score was classified as grade 1 (<10), grade 2 (10–14), or grade 3 (>14) [18]. The ALBI grade was classified as 1 (≤−2.60), 2 (>−2.60 to ≤−1.39), or 3 (>−1.39) [9]. The PALBI grade was classified as 1 (≤−2.53) 2 (>−2.53 to ≤−2.09), or 3 (>−2.09) [15].

**Table 1:** Equation for calculating each grade including CTP score, MELD score, ALBI grade and PALBI grade.

CTP score	Adding points of five categories below		
CTP class	Class A, 5–6 points	Class B, 7–9 points	Class C, 10–15 points
	1 point	2 points	3 points
Albumin (g/dL)	> 3.5	2.8–3.5	< 2.8
Bilirubin (mg/dL)	< 2	2–3	>3
INR	<1.7	1.7–2.3	>2.3
Ascites	None	Mild	Severe
Encephalopathy	None	Grade I or II	Grade III or IV
MELD score	3.78 x log_e_ serum bilirubin (mg/dL) + 11.20 x log_e_ INR + 9.57 x log_e_ serum creatinine (mg/dL) + 6.43		
MELD grade	Grade 1, <10	Grade 2, 10–14	Grade 3, >14
ALBI score	(log_10_ bilirubin × 0.66) + (albumin × -0.085), where bilirubin is in μmol/L and albumin in g/L		
ALBI grade	Grade 1, ≤-2.60	Grade 2, >-2.60 to ≤-1.39	Grade 3, >-1.39
PALBI score	2.02 × log_10_ bilirubin − 0.37 × (log_10_ bilirubin)^2^ − 0.04 × albumin − 3.48 × log_10_ platelets + 1.01 × (log_10_ platelets) ^2^		
PALBI grade	Grade 1, ≤-2.53	Grade 2, >-2.53 to ≤-2.09	Grade 3, >-2.09

CTP, Child-Turcotte-Pugh; INR, International normalized ratio; MELD, Model for end-stage liver disease; ALBI, albumin-bilirubin; PALBI, platelet-albumin-bilirubin

### Tumor staging and treatment group

At the time of HCC diagnosis, tumors were staged using the Barcelona Clinic Liver Cancer (BCLC) and modified Union for Cancer Control staging systems [19, 20]. A multidisciplinary expert group in each hospital decided on the optimum initial treatment plan for each patient according to tumor staging following the BCLC and/or KLCSG-NCC guidelines. LT, surgical resection (SR), radiofrequency ablation (RFA), transarterial chemoembolization (TACE), sorafenib, and supportive-care treatments were administered by experts.

### Statistics

The baseline characteristics of the patients are presented as means ± standard deviations or as counts with percentages, as appropriate [21]. The Kaplan–Meier survival method with the log-rank test was used to assess the CTP class, MELD grade, BCLC stage, ALBI grade, and PALBI grade. Areas under the receiver operating characteristic curve (AUCs) were calculated for the 1-, 3-, and 5-year mortality rates. Harrell’s *c* statistic was also calculated for each grade. The ability of the ALBI and PALBI grades to predict OS stratified by CTP class and treatment modality was evaluated using the Kaplan–Meier method. A multivariate Cox regression analysis was performed to identify risk factors for OS according to treatment modality. In this analysis, model 1 included the ALBI grade, but not the PALBI grade; model 2 included the PALBI grade, but not the ALBI grade; and model 3 included the ALBI and PALBI grades. All statistical analyses were performed by biostatistics team of Catholic university of Korea using SAS version 9.4 (SAS Institute Inc., Cary, NC, USA).

## Results

### Baseline characteristics

The mean age of the patients was 59.7 ± 11.4 years (range, 52.0–68.0 years), and the majority (*n* = 5,144, 79.1%) was male. HBV (63.4%) was the most frequent etiology of HCC, followed by alcohol (22.7%) and HCV (12.5%). The majority (*n* = 4,669, 71.8%) of patients was of CTP class A, and the mean MELD score was 9.8 ± 4.0 (range, 7.0–11.0). The most frequent ALBI grade was 2 (49.3%) and the most frequent PALBI grade was 1 (38.4%), followed by 2 (33.2%).

Most (61.2%) patients had single tumors, and the mean tumor size was 4.8 ± 3.9 cm (range, 2.0–6.3 cm). The largest proportion (40.5%) of patients was of BCLC stage A, followed by stage C (33.2%). The most frequent treatment modality was TACE (45.8%), followed by SR (18.2%), supportive care (17.6%), and RFA (11.6%). The baseline characteristics of the patients are summarized in Table 2.

**Table 2:** Baseline characteristics of included patients.

	All patients (n = 6507)
Age, years	59.7±11.4
gender (male/female)	5144(79.1%)/1363 (20.9%)
Etiologies	
Hepatitis B, n (%)	4036(63.4%)
Hepatitis C, n (%)	778(12.5%)
alcohol, n (%)	1479(22.7%)
non-B, C hepatitis, n (%)	214(3.4%)
Diabetes mellitus, n (%)	1530(23.7%)
Hypertension, n (%)	2027(31.4%)
Laboratory values	
Alpha-fetoprotein^a^, ng/mL, n (%)	12411.7±97615.7
≤20	2542(41.5%)
20–400	1819(29.7%)
>400	1760(28.8%)
PIVKA-II^b^, mAU/mL	3128.1±16406.5
≤40	1155 (33.6%)
>40	2282 (66.4%)
Albumin, g/dL	3.7±0.7
<2.8	586 (9.0%)
2.8–3.5	1893 (29.1%)
>3.5	4028 (61.9%)
Total bilirubin, mg/dL	1.7±3.0
<2	5386 (82.8%)
≥2-≤3	574 (8.8%)
>3	547 (8.4%)
Platelets, 1000/μL	156.4±90.3
Child-Turcotte-Pugh (CTP) Class, n (%)	
A	4669 (71.8%)
B	1522 (23.4%)
C	316 (4.8%)
ALBI score	-2.3±0.7
ALBI grade, n (%)	
grade 1 (≤-2.60)	2575 (39.6%)
grade 2 (>-2.60 to -1.39)	3211 (49.3%)
grade 3 (>-1.39)	721 (11.1%)
PALBI score	-2.3±0.5
PALBI grade	
grade 1 (≤-2.53)	2499 (38.4%)
grade 2 (-2.53 to -2.09)	2158 (33.2%)
grade 3 (>-2.09)	1850 (28.4%)
MELD score	9.8±4.0
MELD grade, n (%)	
grade 1 (<10)	4075 (62.6%)
grade 2 (10 to 14)	1738 (26.7%)
grade 3 (>14)	694 (10.7%)
Tumor number ^c^, n (%)	
1	3981 (61.2%)
2	940 (14.4%)
≥3	1585 (24.4%)
Maximal tumor diameter^d^, cm	4.8±3.9
≤2 cm, n (%)	1734 (29.2%)
2–5 cm, n (%)	2336 (39.3%)
>5 cm, n (%)	1877 (31.5%)
Portal vein invasion, n (%)	1511 (23.2%)
BCLC stages, n (%)	
0	593 (9.1%)
A	2635 (40.5%)
B	718 (11.0%)
C	2156 (33.2%)
D	405 (6.2%)
TNM stages, n (%)	
I	983 (15.1%)
I	2425 (37.3%)
III	1707 (26.2%)
IV-A	760 (11.7%)
IV-B	632 (9.7%)
Initial treatment modalities, n (%)	
Surgical resection	1187 (18.2%)
Liver transplantation	60 (0.9%)
Radiofrequency ablation (RFA)	757 (11.6%)
Trans-arterial chemoembolization (TACE)	2982 (45.8%)
Sorafenib	212 (3.3%)
Radiation therapy	82 (1.3%)
Supportive care	1147 (17.6%)
Systemic chemotherapy	80 (1.2%)

ALBI, albumin-bilirubin; PALBI, platelet-albumin-bilirubin; MELD, model for end-stage liver disease; BCLC stage, Barcelona Clinic Liver Cancer stage; TNM stage, Tumor, Node, Metastasis

^a^n = 386 missing data is not included to analysis;

^b^n = 3070 missing data is not included to analysis;

^c^n = 1 missing data is not included to analysis;

^d^n = 560 missing data is not included to analysis

### OS and AUC according to liver function grade

**Fig 2:**
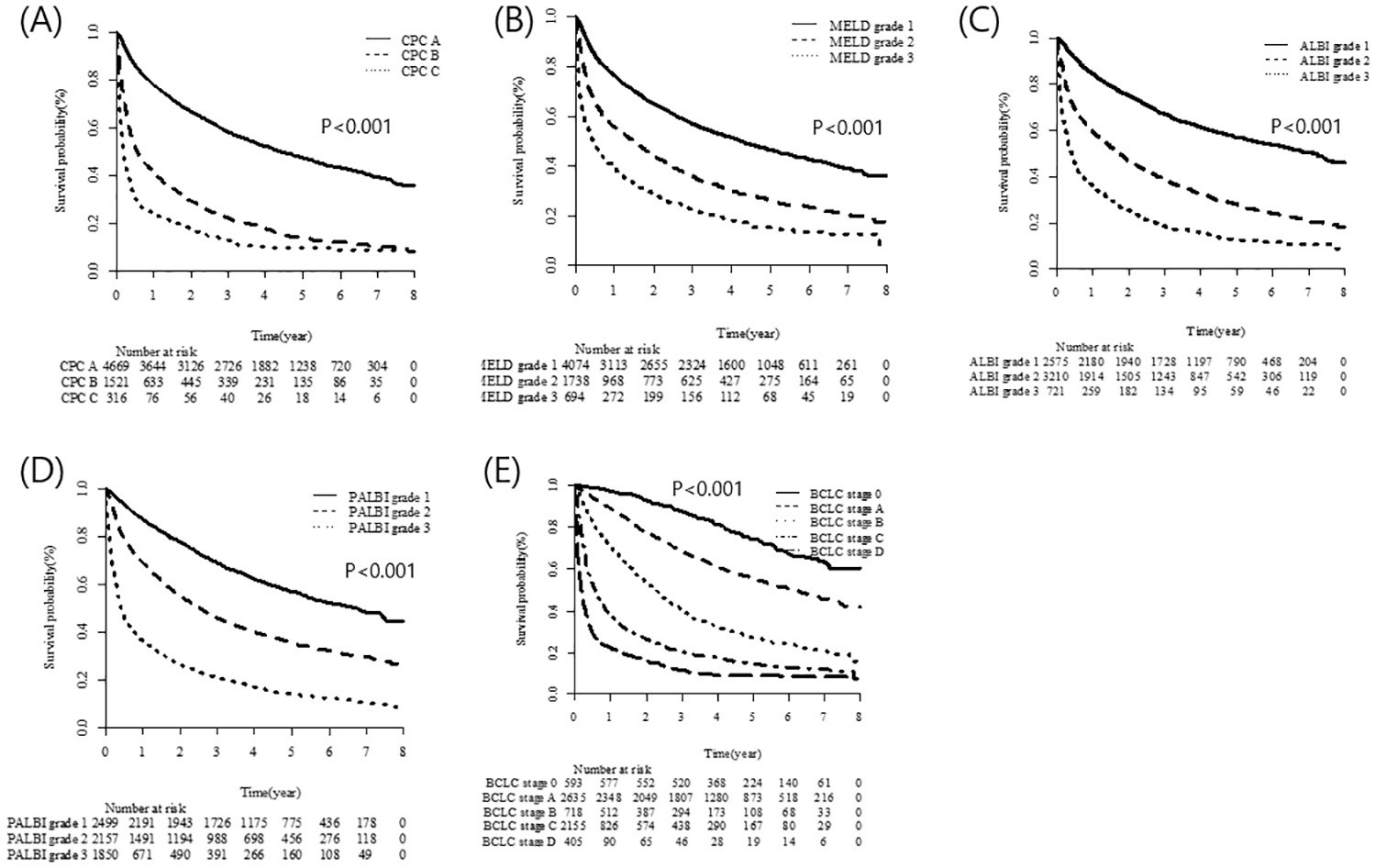
OS stratified by liver function assessment grade and BCLC stage.

The median follow-up period was 32 months (range: 0–95 months) and the median OS of the entire cohort was 32 months (95% Confidence Interval (CI): 30–34 months). The 5-year survival rate of our nationwide cohort was 0.38 (95% CI: 0.36–0.39). OS was stratified according to the various liver function–grading systems (Fig 2). The CTP class, MELD score, ALBI grade, and PALBI grade were associated significantly with OS (P<0.001 for each grade, Fig 2A and 2D). The BCLC stage also enabled stratification of OS (P<0.001, Fig 2E).

Harrell’s *c* statistic for OS was significantly higher for the PALBI grade than for the CTP class, MELD grade, and ALBI grade (0.675 *vs*. 0.633, 0.607, and 0.642, respectively; P<0.001). Moreover, the PALBI grade had the highest AUC values for the 1-, 3-, and 5-year mortality rates (0.750, 0.711, and 0.696, respectively; P<0.001; Table 3).

**Table 3:** AUC value and Harrell’s C-statics for comparing each grades.

	Harrell’s C-statistic (95% CI)	*1-year mortality*			*3-year mortality*			*5-year mortality*		
		AUC (95% CI)	*p* value^a^	p value^b^	AUC (95% CI)	*p* value^a^	*p* value^b^	AUC (95% CI)	*p* value^a^	*p* value^b^
*All patients*										
MELD grade	0.607 (0.599–0.615)	0.645 (0.632–0.658)	reference	<0.001	0.625 (0.613–0.636)	reference	<0.001	0.618 (0.604–0.632)	reference	<0.001
CTP class	0.633 (0.625–0.640)	0.685 (0.673–0.697)	<0.001	<0.001	0.656 (0.646–0.666)	<.0001	<0.001	0.647 (0.636–0.658)	<.0001	<0.001
ALBI grade	0.642 (0.634–0.649)	0.688 (0.676–0.700)	<0.001	<0.001	0.676 (0.664–0.688)	<.0001	<0.001	0.669 (0.654–0.684)	<.0001	<0.001
PALBI grade	0.675 (0.667–0.682)	0.750 (0.738–0.762)	<0.001	reference	0.711 (0.699–0.723)	<.0001	reference	0.696 (0.682–0.711)	<.0001	reference

AUC, The area under the receiver operating characteristics curve; MELD, model for end-stage liver disease; CTP, Child-Turcotte-Pugh; ALBI, albumin-bilirubin; PALBI, platelet-albumin-bilirubin

^a^P value in the table denotes for comparison between MELD with other scores;

^b^P value in the table denotes for comparison between PALBI with other scores

### Stratification of CTP class according to ALBI grade and PALBI grade

**Fig 3:**
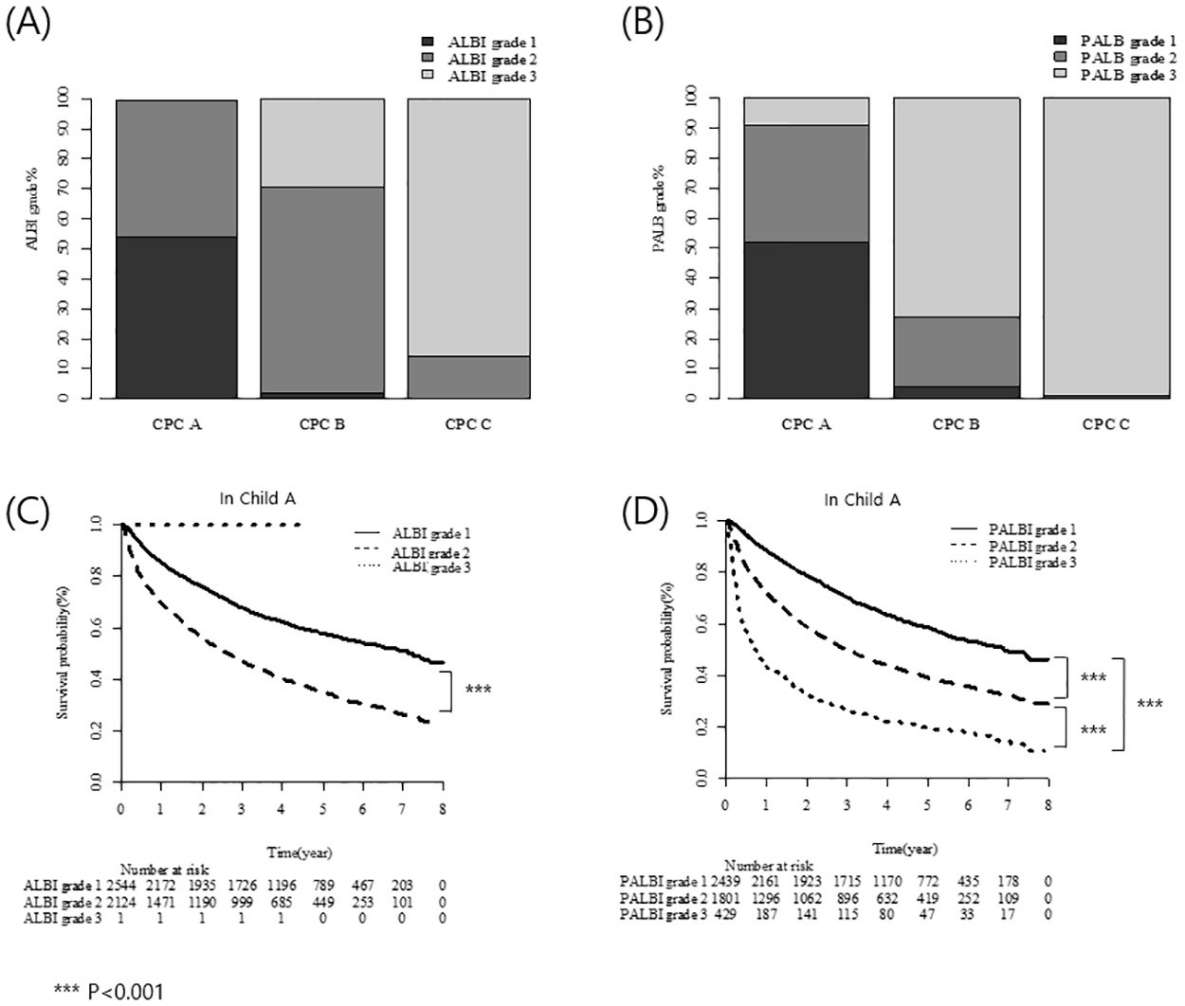
CTP class stratified by ALBI and PALBI grades.

The CTP class was stratified according to the ALBI and PALBI grades. Of the CTP A patients (*n* = 4,669), 54.5% (*n* = 2,544), 45.5% (*n* = 2,124), and 0.02% (*n* = 1) were of ALBI grades 1, 2, and 3, respectively. Of the CTP B patients (*n* = 1,522), 31 (2.0%), 1,042 (68.5%), and 449 (29.5%) were of ALBI grades 1, 2, and 3, respectively. Of the CTP C patients, 14.2% (*n* = 45) and 85.8% (*n* = 271) were of ALBI grades 2 and 3, respectively. No patient was of ALBI grade 1 (Fig 3A).

Of the CTP A patients (*n* = 4,669), 52.2% (*n* = 2,439), 38.6% (*n* = 1,801), and 9.2% (*n* = 429) were of PALBI grades 1, 2, and 3, respectively. Of the CTP B patients (*n* = 1,522), 23.3% (*n* = 60), 23.3% (*n* = 354), and 72.8% (*n* = 1,108) were of PALBI grades 1, 2, and 3, respectively. Of the CTP C patients (*n* = 316), 0% (*n* = 0), 1% (*n* = 3), and 99.1% (*n* = 313) were of PALBI grades 1, 2, and 3, respectively (Fig 3B).

The ALBI and PALBI grades enabled prediction of OS in patients with CTP class A HCC (Fig 3C and 3D). OS was significantly longer for ALBI grade 1 than for ALBI grade 2 patients (median, 86 *vs*. 31.5 months, respectively; *P* < 0.001). PALBI grade 1 patients had the longest OS, followed by those of grades 2 and 3 (median, 83 *vs*. 35 and 8 months, respectively; *P* < 0.001 between each grade). However, the ALBI and PALBI grades were not predictive of OS for CTP B and C patients, with the exception of grade 2 *vs*. 3 in CTP B patients (*P* < 0.001).

### Ability of ALBI and PALBI grades for OS according to different etiologies and level of tumor marker

**Fig 4:**
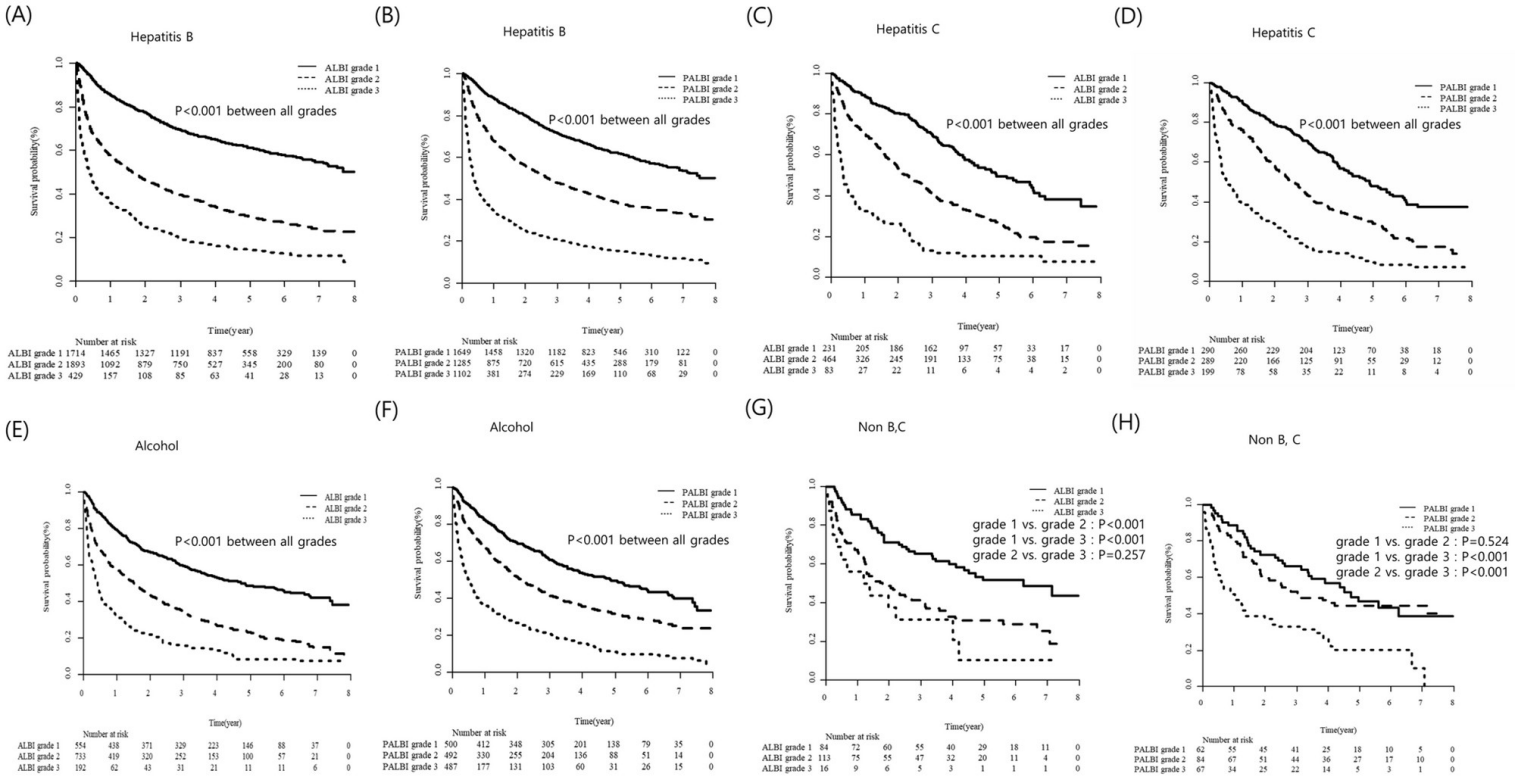
Utility of ALBI and PALBI grades according to different etiologies.

The ability of ALBI and PALBI grade in predicting OS was evaluated according to etiologies (Fig 4). In hepatitis B patients, which is the major etiology of this cohort, both ALBI and PALBI grades were significant in predicting OS by grades (*P* < 0.001 for each grade; Fig 4A and 4B). Both ALBI and PALBI grades are also significantly differentiated OS by grades in patients with hepatitis C (*P* <0.001 for each grade; Fig 4C and 4D) and alcohol (*P* < 0.001 for each grade; Fig 4E and 4F). In Non-B, C patients, ALBI and PALBI grade showed good predictive ability for OS (*P* <0.001), with the exception of ALBI grade 2 vs. grade 3 (*P* = 0.257) and PALBI grade 1 vs. grade 2 (*P* = 0.524; Fig 4G and 4H).

**Fig 5:**
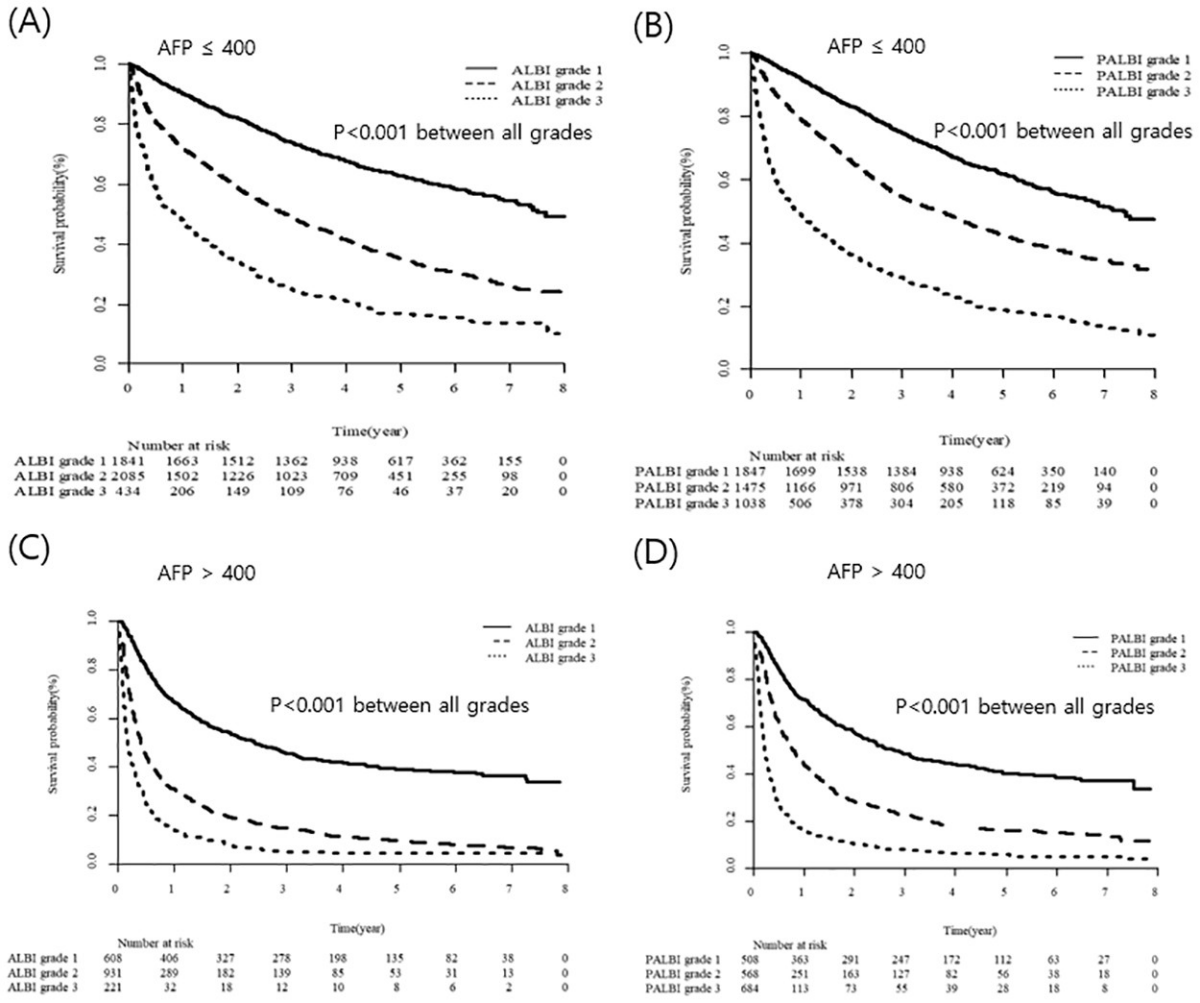
Utility of ALBI and PALBI grades according to tumor marker.

We also investigated the predictive value of ALBI and PALBI grades for OS according to the level of AFP (Fig 5). The cut off level of AFP (400 ng/mL) was classified into low AFP (AFP ≤ 400 ng/mL) and high AFP group (AFP > 400 ng/mL) [22, 23]. In low AFP group, both ALBI and PALBI grade could stratified OS by their grades (*P* < 0.001 for each grade; Fig 5A and 5B). Moreover, in high AFP group, OS was significantly differentiated by both ALBI and PALBI grades (*P* < 0.001 for each grade; Fig 5C and 5D).

### Predictive power of ALBI and PALBI grades according to treatment modality

**Fig 6:**
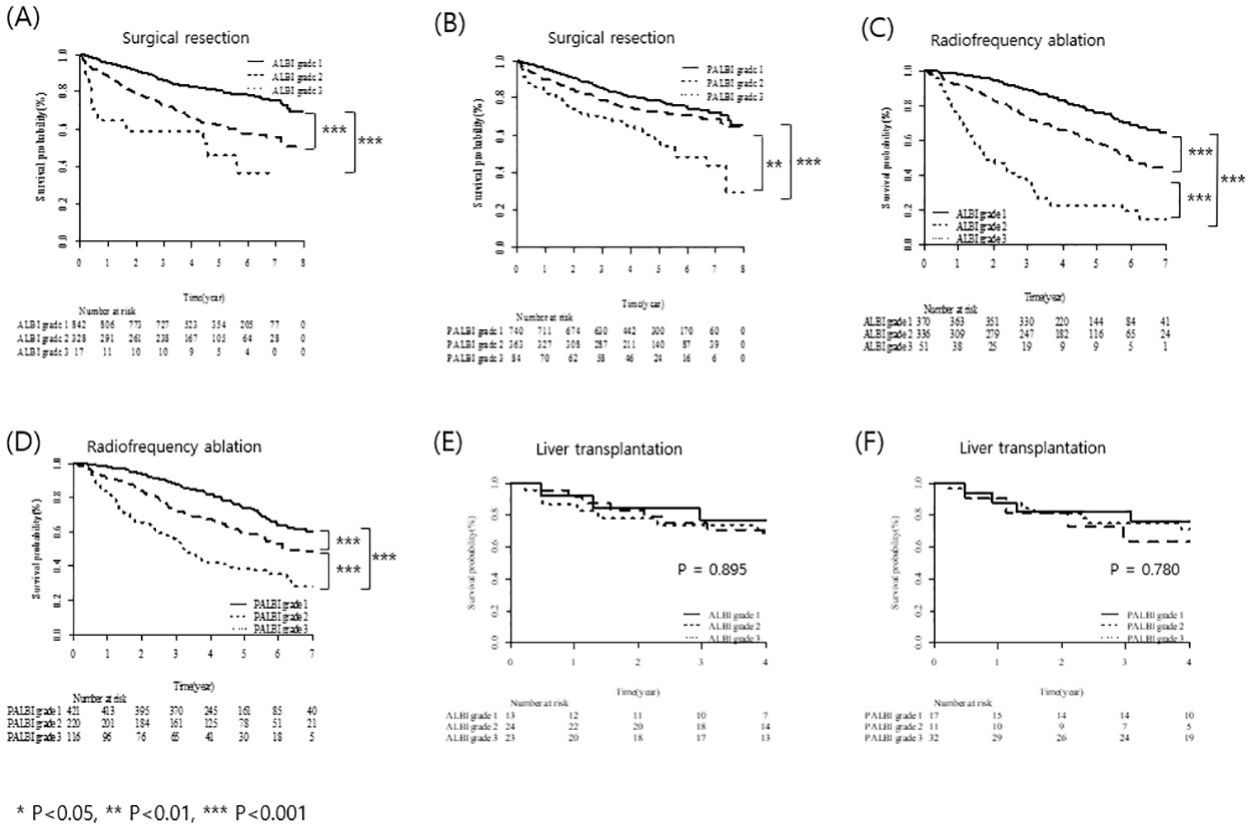
Utility of the ALBI and PALBI grades according to curative treatment modality.

The predictive power of the ALBI and PALBI grades for OS was assessed according to curative treatment modality (SR, RFA, and LT; Fig 6). In patients undergoing SR, the ALBI grade was significantly predictive of OS, with the exception of grade 2 *vs*. 3 (grade 1 *vs*. 2 and 3, *P* < 0.001; grade 2 *vs*. 3, *P* = 0.230; Fig 6A). The PALBI grade also significantly differentiated OS by grades, with the exception of grade 1 *vs*. 2 (grade 1 *vs*. 2, *P* = 0.107; grade 2 *vs*. 3, *P* < 0.05; grade 3 *vs*. 1, *P* < 0.001; Fig 6B). The ALBI and PALBI grades showed good predictive performance for OS in patients receiving RFA (*P* < 0.001 for each grade; Fig 6C and 6D). However, in patients receiving LT, the ALBI and PALBI grades were not predictive of OS (ALBI grade, *P* = 0.895; PALBI grade, *P* = 0.780; Fig 6E and 6F).

**Fig 7:**
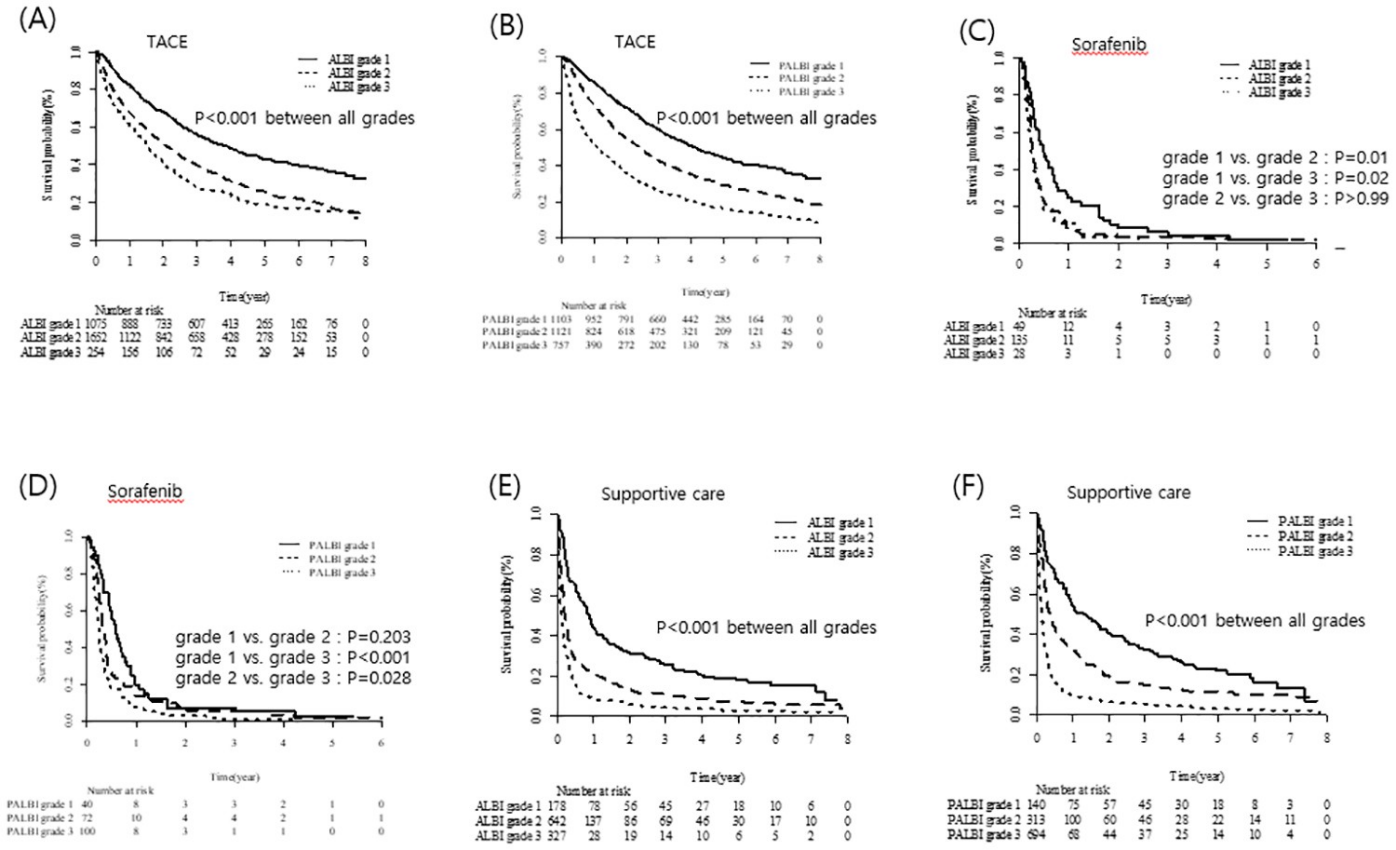
Utility of the ALBI and PALBI grades according to palliative treatment modality.

The predictive power of the ALBI and PALBI grades for OS was next assessed according to palliative treatment modality (TACE, sorafenib, and supportive care). In patients receiving TACE, the ALBI and PALBI grades were significantly predictive of OS (*P* < 0.001 for each grade; Fig 7A and 7B). In patients on sorafenib, the ALBI grade was significantly predictive of OS, with the exception of grade 2 *vs*. 3 (grade 1 *vs*. 2, *P* = 0.01; grade 1 *vs*. 3, *P* = 0.02; grade 2 *vs*. 3, *P* > 0.99; Fig 7C). OS differed significantly between PALBI grades 1 and 2 *vs*. 3 (*P* < 0.001 and 0.028, respectively). However, OS did not differ between PALBI grades 1 and 2 in the sorafenib group (*P* = 0.203; Fig 7D). In patients on supportive care, the ALBI and PALBI grades were significantly predictive of OS (*P* < 0.001 for each grade; Fig 7E and 7F).

### Analysis of OS according to treatment modality with BCLC stage

The results of univariate and multivariate Cox regression analyses for OS are shown in Table 4. Age, male sex, Non-B&C, maximum tumor diameter, AFP level, and CTP class were independent risk factors for OS. The ALBI and PALBI grades were associated significantly with OS in the multivariate analysis.

**Table 4:** Cox regression analysis including ALBI and PALBI grade on overall survival.

	Univariate analysis		Multivariate analysis	
	Hazard ratio (95% CI)	p value	Hazard ratio (95% CI)	p value
Age, years	1.01 (1.01–1.02)	<0.001	1.02 (1.01–1.02)	<0.001
Gender (Female vs. male)	0.84 (0.77–0.90)	<0.001	0.83 (0.76–0.91)	<0.001
CTP Class				
Class B vs. Class A	2.91 (2.72–3.12)	<0.001	1.91(1.72–2.13)	<0.001
Class C vs. Class A	4.66 (4.12–5.27)	<0.001	3.27 (2.66–4.01)	<0.001
Etiology				
Hepatitis B	0.81 (0.76–0.87)	<0.001	1.04 (0.79–1.36)	0.802
Hepatitis C	1.11 (1.01–1.22)	0.024	1.15 (0.89–1.49)	0.277
Non-B, C	0.94 (0.79–1.11)	0.464	0.71 (0.51–0.99)	0.043
Alcohol	1.27 (1.18–1.36)	<0.001	1.16 (0.88–1.54)	0.297
Maximal tumor diameter				
2–5 cm vs. ≤2 cm	1.43 (1.30–1.56)	<0.001	1.41 (1.28–1.56)	<0.001
>5 cm vs. ≤2 cm	4.16 (3.81–4.55)	<0.001	3.72 (3.36–4.12)	<0.001
Alpha-fetoprotein, ng/mL				
> 400 vs. ≤ 400	2.43 (2.27–2.59)	<0.001	1.74 (1.61–1.89)	<0.001
ALBI grade				
grade 2 vs. grade 1	2.39 (2.23–2.57)	<0.001	1.70 (1.53–1.89)	<0.001
grade 3 vs. grade 1	4.43 (4.02–4.89)	<0.001	1.56 (1.30–1.86)	<0.001
PALBI grade				
grade 2 vs. grade 1	1.89(1.75–2.05)	<0.001	1.13 (1.02–1.26)	0.023
grade 3 vs. grade 1	4.31(3.98–4.65)	<0.001	1.57 (1.37–1.80)	<0.001

CTP, Child-Turcotte-Pugh; ALBI, albumin-bilirubin; PALBI, platelet-albumin-bilirubin

The patients were evaluated according to initial treatment modality and BCLC stage. First, predictive factors for OS in patients of BLCC stage 0 or A undergoing curative treatment were evaluated (Table 5). Among the 591 patients receiving RFA, 390 survived and 201 died. In the multivariate analysis, the ALBI grade was an independent predictive factor for OS in model 1 (grade 2 *vs*. 1, *P* = 0.009; grade 3 *vs*. 1, *P* = 0.002). However, the PALBI grade was not a significant factor in patients receiving RFA. In model 3, the ALBI grade was an independent predictive factor for OS (grade 2 *vs*. 1, *P* = 0.038; grade 3 *vs*. 1, *P* = 0.001). Among the 855 patients undergoing SR, 672 survived and 183 died. The ALBI grade was a significant factor for OS in the multivariate analysis (model 1, grade 2 *vs*. 1, *P* < 0.001). However, only seven ALBI grade 3 patients underwent SR, and there was no significance between ALBI grades 3 and 1 (model 1; *P* = 0.705). The PALBI grade was not a significant risk factor in model 2. In model 3, the ALBI and PALBI grades had significance between grade 2 *vs*. 1 (*P* < 0.001 and 0.015, respectively).

**Table 5:** Multivariate cox regression analysis on survival according to curative treatment modalities with BCLC stage.

Initial treatment modality	Number of patients		Univariate analysis		Multivariate analysis					
					Model 1		Model 2		Model 3	
	Alive	Dead	crude HR (95% CI)	p value	adjust HR (95% CI)	p value	adjust HR (95% CI)	p value	adjust HR (95% CI)	p value
***RFA patients & BCLC 0*,*A (n = 591*,*alive = 390*,*death = 201)***										
Age, years (mean age)	58.7±.9	62.8±10.9	1.04(1.02–1.05)	<0.001	1.04(1.02–1.05)	<0.001	1.03(1.01–1.05)	0.001	1.03(1.02–1.05)	<0.001
Gender (Female vs. male)	119/307	59/166	0.93(0.69–1.26)	0.651	0.78(0.55–1.10)	0.152	0.83(0.59–1.16)	0.273	0.80(0.57–1.13)	0.208
CTP Class (Class B vs. Class A)	33/393	59/166	3.26(2.42–4.39)	<0.001	2.28(1.53–3.42)	<0.001	3.52(2.23–5.57)	<0.001	3.11(1.93–5.00)	<0.001
Etiology										
Hepatitis B	283	107	0.49(0.38–0.64)	<0.001	0.74(0.25–2.16)	0.576	0.75(0.26–2.19)	0.602	0.79(0.27–2.30)	0.658
Hepatitis C	63	54	1.63(1.19–2.22)	0.002	1.22(0.43–3.51)	0.709	1.23(0.43–3.51)	0.694	1.26(0.44–3.60)	0.666
NBNC	16	4	0.47(0.18–1.28)	0.140	0.32(0.07–1.49)	0.147	0.30(0.07–1.40)	0.126	0.31(0.07–1.44)	0.136
Alcohol	68	58	1.82(1.35–2.445)	<0.001	1.16(0.37–3.58)	0.800	1.20(0.39–3.70)	0.749	0.35(0.04–2.98)	0.339
Maximal tumor diameter										
2–5 cm vs. ≤2 cm	105/319	82/141	1.48(1.13–1.95)	0.005	1.41(1.05–1.89)	0.022	1.45(1.09–1.95)	0.012	1.40(1.05–1.87)	0.024
>5 cm vs. ≤2 cm	2/319	2/141	1.74(0.43–7.04)	0.436	1.53(0.21–11.29)	0.676	1.22(0.17–8.96)	0.843	1.35(0.18–9.90)	0.771
Alpha-fetoprotein, ng/mL										
> 400 vs. ≤ 400	25/273	15/192	1.31 (0.77–2.22)	0.315	1.93(1.10–3.38)	0.021	1.79(1.03–3.13)	0.04	1.95(1.11–3.40)	0.019
ALBI grade										
grade 2 vs. grade 1	169/251	127/81	1.95(1.48–2.58)	<0.001	1.55(1.12–2.14)	0.009			1.55(1.02–2.35)	0.038
grade 3 vs. grade 1	6/251	17/81	5.10(3.02–8.60)	<0.001	3.05(1.53–6.08)	0.002			4.27(1.89–9.66)	<0.001
PALBI grade										
grade 2 vs. grade 1	116/277	80/105	1.56(1.17–2.09)	0.003			1.37(0.99–1.90)	0.059	1.05(0.69–1.59)	0.815
grade 3 vs. grade 1	33/277	40/105	2.41(1.67–3.47)	<0.001			0.93(0.52–1.67)	0.806	0.51(0.25–1.05)	0.068
***Surgical resection & BCLC 0*,*A (n = 855*,*alive = 672*,*death = 183)***										
Age, years	56.1±10.3	58.2±11.2	1.02(1.01–1.04)	0.004	1.01(1.00–1.03)	0.099	1.02(1.00–1.03)	0.058	1.01(1.00–1.03)	0.088
Gender (Female vs. male)	139/572	37/159	0.97(0.68–1.38)	0.845	0.85(0.58–1.26)	0.427	0.85(0.58–1.26)	0.427	0.79(0.53–1.17)	0.242
CTP Class‡ (Class B vs. Class A)	14/697	14/182	3.03(1.76–5.21)	<0.001	1.92(0.91–4.04)	0.086	2.21(1.02–4.79)	0.044	1.84(0.82–4.11)	0.137
Etiology										
Hepatitis B	526	130	0.78(0.57–1.05)	0.102	1.00(0.67–1.35)	0.781	1.01(0.71–1.44)	0.943	0.95(0.67–1.36)	0.789
Hepatitis C	38	25	2.19(1.44–3.34)	<0.001	1.65(0.56–4.87)	0.361	2.02(0.70–5.85)	0.196	1.40(0.45–4.31)	0.559
NBNC	17	2	0.41(0.10–1.64)	0.207	0.32(0.06–1.80)	0.194	0.30(0.05–1.70)	0.176	0.29(0.05–1.71)	0.170
Alcohol	132	39	1.10(0.77–1.56)	0.602	0.95(0.29–3.06)	0.926	0.95(0.30–3.04)	0.927	0.83(0.25–2.81)	0.765
Maximal tumor diameter										
2–5 cm vs. ≤2 cm	415/185	100/35	1.23(0.84–1.81)	0.292	1.23(0.82–1.83)	0.294	1.25(0.83–1.87)	0.281	1.24(0.83–1.86)	0.294
>5 cm vs. ≤2 cm	110/185	59/35	2.39(1.57–3.63)	<0.001	2.53(1.60–3.99)	<0.001	2.48(1.57–3.92)	0.000	2.40(1.52–3.77)	<0.001
Alpha-fetoprotein, ng/mL										
> 400 vs. ≤ 400	122/552	39/148	1.19(0.83–1.70)	0.350	1.18(0.80–1.75)	0.400	1.13(0.76–1.67)	0.555	1.19(0.80–1.76)	0.390
ALBI grade										
grade 2 vs. grade 1	155/553	84/108	2.44(1.83–3.24)	<0.001	2.16(1.58–2.95)	<0.001			2.76(1.89–4.04)	<0.001
grade 3 vs. grade 1	3/553	4/108	4.20(1.55–11.40)	0.005	1.37(0.27–6.86)	0.705			1.64(0.32–8.52)	0.555
PALBI grade										
grade 2 vs. grade 1	201/487	61/114	1.22(0.89–1.67)	0.209			1.04(0.75–1.45)	0.807	0.62(0.42–0.91)	0.015
grade 3 vs. grade 1	23/487	21/114	2.91(1.83–4.64)	<0.001			1.58(0.84–2.97)	0.160	0.83(0.43–1.62)	0.584

RFA, radiofrequency ablation; BCLC, Barcelona clinic liver cancer stage; CTP, Child-Turcotte-Pugh; ALBI, albumin-bilirubin; PALBI, platelet-albumin-bilirubin

The factors predictive of OS according to palliative treatment modality are listed in Table 6. Among the 1,715 patients of BCLC stage 0 to B receiving TACE, 684 survived and 1,030 died. The PALBI grade was a significant risk factor for OS (model 2: grade 2 *vs*. 1, *P* < 0.001; grade 3 *vs*. 1, *P* < 0.001). In model 3, PALBI grade 3 *vs*. grade 1 had significant difference (*P* < 0.001), but there was no significant difference between PALBI grades 2 and 1 (*P* = 0.053). Only ALBI grade 2 *vs*. 1 was a risk factor for OS (model 1, *P* < 0.001; model 3, *P* < 0.001). Of the patients of BCLC stage C on sorafenib (*n* = 111), 4 survived and 107 died. The ALBI and PALBI grades were not predictive of OS in patients on sorafenib. Among the patients on supportive care (*n* = 1,147; 81 survived, 1,066 died), the ALBI and PALBI grades were effective factor for survival in models 1 and 2, respectively. According to model 3, the predictive power of the PALBI grade for OS was superior to that of the ALBI grade (PALBI grade 2 *vs*. 1, *P* = 0.002; grade 3 *vs*. 1, *P* < 0.001).

**Table 6:** Multivariate cox regression analysis on survival according to palliative treatment modalities with BCLC stage.

Initial treatment modality	Number of patients		Univariate analysis		Multivariate analysis					
					Model 1		Model 2		Model 3	
	Alive	Dead	crude HR (95% CI)	p value	adjust HR (95% CI)	p value	adjust HR (95% CI)	p value	adjust HR (95% CI)	p value
***TACE patients& BCLC 0*,*A*,*B (n = 1715*,*alive = 684*,*death = 1030)***										
Age, years	59.7±9.6	62.8±10.8	1.02(1.02–1.03)	<0.001	1.02(1.01–1.03)	<0.001	1.02(1.02–1.03)	<0.001	1.02(1.02–1.03)	<0.001
Gender (Female vs. male)	178/572	262/898	0.94(0.82–1.08)	0.385	0.85(0.73–0.99)	0.034	0.89(0.76–1.03)	0.118	0.86(0.74–1.00)	0.043
CTP Class (Class B vs. Class A)	78/672	288/872	1.87(1.64–2.14)	<0.001	1.82(1.53–2.17)	<0.001	1.43(1.17–1.75)	0.001	1.43(1.17–1.75)	0.001
Etiology										
Hepatitis B	504	622	0.70(0.62–0.79)	<0.001	0.85 (0.54–1.33)	0.480	0.81 (0.51–1.27)	0.355	0.84 (0.53–1.33)	0.457
Hepatitis C	87	196	1.24 (1.07–1.45)	0.006	0.94 (0.60–1.46)	0.772	0.90 (0.57–1.42)	0.655	0.91 (0.58–1.44)	0.696
NBNC	24	35	0.98 (0.70–1.37)	0.913	0.66 (0.38–1.15)	0.144	0.57 (0.32–1.01)	0.054	0.60 (0.34–1.05)	0.074
Alcohol	135	299	1.37 (1.20–1.57)	<0.001	0.98 (0.62–1.57)	0.943	0.90 (0.56–1.45)	0.663	0.95 (0.59–1.52)	0.824
Maximal tumor diameter										
2–5 cm vs. ≤2 cm	329/343	522/331	1.45(1.27–1.67)	<0.001	1.37(1.18–1.58)	<0.001	1.36(1.17–1.57)	<0.001	1.38(1.19–1.60)	<0.001
>5 cm vs. ≤2 cm	77/343	270/331	2.67(2.28–3.14)	<0.001	2.65(2.21–3.17)	<0.001	2.53(2.12–3.03)	<0.001	2.64(2.20–3.16)	<0.001
Alpha-fetoprotein, ng/mL										
> 400 vs. ≤ 400	88/611	227/867	1.61 (1.38–1.87)	<0.001	1.45 (1.23–1.70)	<0.001	1.40 (1.20–1.65)	<0.001	1.41 (1.20–1.66)	<0.001
ALBI grade										
grade 2 vs. grade 1	332/395	690/385	1.62(1.43–1.83)	<0.001	1.55(1.34–1.79)	<0.001			1.37(1.15–1.62)	<0.001
grade 3 vs. grade 1	23/395	85/385	2.26(1.79–2.86)	<0.001	1.39(1.02–1.90)	0.036			1.00(0.72–1.40)	0.992
PALBI grade										
grade 2 vs. grade 1	250/428	469/424	1.51(1.32–1.72)	<0.001			1.38(1.20–1.60)	<0.001	1.18(1.00–1.39)	0.053
grade 3 vs. grade 1	72/428	267/424	2.32(1.99–2.70)	<0.001			2.09(1.67–2.62)	<0.001	1.86(1.46–2.38)	<0.001
***sorafenib & BCLC C (n = 111*, *alive = 4*, *death = 107)***										
Age, years	62.0±7.4	55.5±11.5	0.99(0.98–1.00)	0.086	0.99(0.97–1.01)	0.402	0.99(0.97–1.02)	0.436	0.99(0.96–1.01)	0.288
Gender (Female vs. male)	0/4	23/150	1.15(0.74–1.79)	0.524	1.45(0.73–2.88)	0.296	1.47(0.73–2.95)	0.285	1.49(0.74–3.03)	0.266
CTP Class (Class B vs. Class A)	0/4	75/98	1.91(1.41–2.60)	<0.001	2.32(1.44–3.73)	0.001	1.95(1.20–3.19)	0.007	2.04(1.24–3.37)	0.005
Etiology										
Hepatitis B	3	123	1.28(0.91–1.80)	0.157	2.17 (0.71–6.62)	0.175	2.09 (0.72–6.10)	0.178	2.12 (0.70–6.40)	0.185
Hepatitis C	0	18	0.83 (0.51–1.36)	0.455	1.67 (0.57–4.90)	0.348	1.62 (0.59–4.46)	0.349	1.63 (0.57–4.69)	0.362
NBNC	0	8	0.89 (0.43–1.80)	0.736	2.42 (0.51–11.4)	0.265	2.36 (0.52–10.66)	0.263	2.57 (0.54–12.18)	0.236
Alcohol	1	27	0.85 (0.57–1.29)	0.45	1.27 (0.38–4.31)	0.699	1.03 (0.32–3.32)	0.961	1.07 (0.32–3.57)	0.919
Maximal tumor diameter										
2–5 cm vs. ≤2 cm	0/1	15/3	4.10(1.16–14.40)	0.028	3.59(0.94–13.7)	0.062	3.27(0.82–13.02)	0.093	3.16(0.80–12.51)	0.102
>5 cm vs. ≤2 cm	1/3	95/3	2.67(0.84–8.49)	0.097	2.44(0.70–8.46)	0.160	2.04(0.58–7.22)	0.267	1.99(0.56–7.06)	0.290
Alpha-fetoprotein, ng/mL										
> 400 vs. ≤ 40	3/1	104/63	1.54 (1.05–2.25)	0.029	1.63 (1.08–2.46)	0.020	1.68 (1.12–2.54)	0.013	1.68 (1.11–2.53)	0.014
ALBI grade										
grade 2 vs. grade 1	3/1	119/40	1.58(1.10–2.27)	0.013	1.38(0.77–2.49)	0.280			1.20(0.63–2.30)	0.579
grade 3 vs. grade 1	0/1	14/40	2.55(1.37–4.73)	0.003	1.25(0.50–3.17)	0.632			0.81(0.30–2.20)	0.683
PALBI grade										
grade 2 vs. grade 1	2/1	60/36	1.44(0.95–2.18)	0.088			1.10(0.59–2.07)	0.764	0.99(0.50–1.97)	0.986
grade 3 vs. grade 1	1/1	77/36	2.35(1.57–3.52)	<0.001			1.95(1.05–3.64)	0.036	1.89(0.96–3.72)	0.064
***supportive patients (n = 1147*, *alive = 81*, *death = 1066)***										
Age, years	59.3±11.8	62.9±12.6	1.00(0.99–1.00)	0.200	1.01(1.00–1.01)	0.051	1.01(1.00–1.01)	0.043	1.01(1.00–1.01)	0.041
Gender (Female vs. male)	19/62	206/860	0.91(0.78–1.06)	0.232	0.97(0.81–1.17)	0.752	0.97(0.81–1.17)	0.748	0.96(0.80–1.16)	0.670
CTP Class										
Class B vs. Class A	20/61	474/400	2.03(1.77–2.32)	<0.001	1.74(1.43–2.11)	<0.001	1.61(1.32–1.95)	<0.001	1.53(1.24–1.89)	<0.001
Class C vs. Class A	0/61	192/400	3.41(2.85–4.07)	<0.001	3.09(2.30–4.14)	<0.001	3.03(2.36–3.94)	<0.001	2.68(1.98–3.63)	<0.001
Etiology										
Hepatitis B	39	572	1.22(1.08–1.38)	0.002	0.95 (0.61–1.46)	0.798	0.96 (0.63–1.48)	0.861	0.95 (0.62–1.47)	0.824
Hepatitis C	3	131	0.93 (0.77–1.12)	0.427	0.94 (0.63–1.42)	0.773	0.96 (0.64–1.45)	0.838	0.96 (0.64–1.45)	0.842
NBNC	1	24	0.91 (0.60–1.36)	0.629	0.95 (0.50–1.80)	0.880	0.91 (0.48–1.71)	0.767	0.90 (0.48–1.70)	0.754
Alcohol	35	340	0.86 (0.75–0.97)	0.018	0.96 (0.61–1.51)	0.867	0.96 (0.61–1.52)	0.871	0.96 (0.61–1.51)	0.850
Maximal tumor diameter										
2–5 cm vs. ≤2 cm	32/30	217/93	1.47(1.16–1.88)	0.002	1.51(1.16–1.96)	0.002	1.44(1.11–1.87)	0.007	1.46(1.12–1.90)	0.005
>5 cm vs. ≤2 cm	16/30	516/93	2.87(2.29–3.59)	<0.001	3.23(2.51–4.15)	<0.001	2.96(2.31–3.79)	<0.001	3.08(2.39–3.96)	<0.001
Alpha-fetoprotein, ng/mL										
> 400 vs. ≤ 400	14/57	463/522	2.13(1.82–2.49)	<0.001	1.50 (1.28–1.75)	<0.001	1.49 (1.28–1.74)	<0.001	1.50 (1.28–1.75)	<0.001
ALBI grade										
grade 2 vs. grade 1	43/30	599/258	1.83(1.53–2.19)	<0.001	1.43(1.14–1.79)	0.002			1.00(0.76–1.32)	0.985
grade 3 vs. grade 1	8/30	463/258	2.89(2.38–3.53)	<0.001	1.88(1.38–2.56)	<0.001			1.21(0.84–1.73)	0.308
PALBI grade										
grade 2 vs. grade 1	34/28	279/112	1.58(1.27–1.97)	<0.001			1.60(1.24–2.07)	0.000	1.60(1.20–2.15)	0.002
grade 3 vs. grade 1	19/28	675/112	3.18(2.59–3.89)	<0.001			2.22(1.69–2.91)	<0.001	2.16(1.55–2.99)	<0.001

TACE, transarterial chemoembolization; BCLC, Barcelona clinic liver cancer stage; CTP, Child-Turcotte-Pugh; ALBI, albumin-bilirubin; PALBI, platelet-albumin-bilirubin

## Discussion

In this analysis of nationally representative data, we evaluated the ability of various scoring system focused on the ALBI and PALBI grades to predict the OS of patients with HCC. The ALBI and PALBI grades had higher AUC values than did the CTP class and MELD score. The ALBI and PALBI grades also showed good predictive performance for OS in patients with HCC. Moreover, each grade has different strength according to treatment modalities. Therefore, the ALBI and PALBI grades may be used to assess liver function and predict the survival of patients with HCC according to treatment modalities.

Our analysis showed that the PALBI and ALBI grades enable prediction of OS. In patients with HCC of CTP class A, the PALBI and ALBI grades enabled discrimination of OS during the 5-year study period. The majority (71.8%) of patients with HCC were of CTP class A. Patients with CTP class A HCC can have various clinical courses, including no chronic liver disease, chronic inflammation only, and well-compensated cirrhosis [24, 25]. Although the CTP class has been used to estimate the liver functional reserve and predict OS in patients with HCC, the subclassification of same CTP class A enables prediction of OS in patients with HCC with well-preserved liver function. Thus, the PALBI and ALBI grades allow assessment of the liver functional reserve.

In the view of etiologies and tumor marker, both ALBI and PALBI grades could predict and stratify OS across all different etiologies and the level of tumor marker. However, because non-B,C patients are only small number in our study with possible heterogeneous cause of hepatitis, it may affect the negative result in the stratification of OS between ALBI grade 2 vs.3 and PALBI grade 1 vs. 2. In multivariate analysis, the ALBI and PALBI grade are independently predictive factor for OS regardless of etiologies and AFP level. Therefore, the PALBI and ALBI grades could be applied to predict OS irrespective of etiology and tumor marker level.

We also evaluated the ability of the ALBI and PALBI grades to predict OS according to BCLC stage and treatment modality. First, we investigated both grading systems according to curative treatment modalities. ALBI grades 1 and 2, but not the PALBI grade, were predictive of OS of patients with HCC undergoing SR. The lack of a difference between ALBI grades 2 and 3 may be due to the small number of ALBI grade 3 patients undergoing SR (*n* = 7). The OS of patients undergoing RFA differed significantly among ALBI grades 1 to 3, but not according to the PALBI grade. These results are in agreement with previous reports that the ALBI grade is predictive of survival after liver resection and RFA [26, 27]. However, the ALBI grade showed greater predictive power than the PALBI grade in this study. Patients undergoing curative treatments typically have good liver function and relatively high platelet counts [28, 29], which may explain our finding of greater predictive power in patients with HCC receiving curative treatments. In contrast, neither the ALBI grade nor the PALBI grade was predictive of OS in patients with HCC receiving LT. The ALBI and PALBI grades predict the risk of mortality related to underlying liver dysfunction. OS prediction using these grades in patients receiving LT may be difficult because LT may not only treat HCC, but also manage underlying liver dysfunction, and because many other factors affect survival during the perioperative period [30].

Second, we evaluated the predictive power for OS of the ALBI and PALBI grades according to palliative treatment modality. In patients with HCC receiving TACE, PALBI grades 1 to 3, and ALBI grades 1 to 2, were significantly predictive of OS. Similarly, the ALBI and PALBI grades are reportedly predictive of survival in patients with HCC undergoing TACE [27, 31]. In our study, ALBI grades 2 and 3 were not significantly predictive of OS. Some ALBI grade 3 patients treated with TACE had severe portal hypertension, resulting in reduced platelet counts, which cannot be distinguished using the ALBI grade. This limitation may have contributed to the negative results for ALBI grades 2 and 3. In patients with HCC of BCLC stage C on sorafenib, PALBI grades 1 and 3, but not the ALBI grades, were significantly predictive of OS. In model 3, both ALBI and PALBI grades are not significant factor of OS. The high risk of mortality of patients of these patients with sorafenib may be related to tumor progression, rather than underlying liver dysfunction. The OS of patients receiving supportive care differed significantly according to the ALBI grade and to PALBI grades 1 to 3. The PALBI grade had greater predictive power than the ALBI grade. Therefore, the evaluation of underlying liver function, including a surrogate for portal hypertension, is important, particularly in patients on supportive treatment. Therefore, the PALBI grade may enable more refined discrimination among patients with HCC receiving palliative treatments; this issue warrants further investigation.

This study has several strengths and limitations. First, we used Korean HCC registry data for a 5-year period. We evaluated a nationally representative cohort of patients with HCC, increasing the accuracy of the results. Second, our results highlight the importance of identifying candidates using more suitable grade system according to BCLC staging and treatment. Although other authors have demonstrated the utility of the ALBI and PALBI grades, these grades have not been studied to identify candidates for whom particular treatment modalities are suitable. Our results suggest that the ALBI grade is more useful than the PALBI grade in patients with HCC undergoing curative treatment, and *vice versa* for those receiving palliative treatment. Third, we analyzed data of treatment modality with BCLC stage. The treatment decision-making process differed among the participating centers, likely reflecting clinical practice, in which various factors are considered before making decision. To reduce bias, we defined treatment modality with BCLC stage. Forth, unfortunately, there was no data about antiviral therapy in HCC patients with HBV and HCV in our cohort. However, as Korean medical insurance covered HCC patients for antiviral treatment, HCC patients with HBV received and maintained antiviral treatment according to HCC guideline. In HCV treatment, Direct Acting Antivirals (DAA) therapy was not available in Korea during the study period. So, HCC patients with HCV did not receive DAA.

In conclusion, the ALBI and PALBI grades showed good performance for the assessment of liver function in patients with HCC. The ALBI grade showed greater predictive power for OS than did the PALBI grade in patients with HCC receiving curative treatment, and the opposite was true for those receiving palliative treatment. Therefore, appropriate use of the ALBI and PALBI grades will enable more-accurate prediction of the prognosis of patients with HCC.

## Abbreviations

AFP: alpha-fetoprotein
ALBI: albumin-bilirubin
AUC: area under the receiver operator characteristic curve
BCLC: Barcelona Clinic Liver Cancer
CTP: Child-Turcotte-Pugh
DAA: Direct Acting Antivirals
HBV: hepatitis B virus
HCC: hepatocellular carcinoma
HCV: hepatitis C virus
KCCR: Korean Central Cancer Registry
KLCA: The Korean Liver Cancer Association
LT: liver transplantation
MELD: Model for End-stage Liver Disease
MRI: magnetic resonance imaging
OS: overall survival
PALBI: Platelet-albumin-bilirubin
RFA: radiofrequency ablation
SR: surgical resection
TACE: transarterial chemoembolization
